# Redetermination of durangite, NaAl(AsO_4_)F

**DOI:** 10.1107/S160053681204384X

**Published:** 2012-10-27

**Authors:** Gordon W. Downs, Betty N. Yang, Richard M. Thompson, Michelle D. Wenz, Marcelo B. Andrade

**Affiliations:** a University High School, 421 N. Arcadia Avenue, Tucson, Arizona 85711-3032, USA; b Catalina Foothills High School, 4300 E. Sunrise Drive, Tucson, Arizona 85718-4300, USA; c Department of Geosciences, University of Arizona, 1040 E. 4th Street, Tucson, Arizona 85721-0077, USA

## Abstract

The crystal structure of durangite, ideally NaAl(AsO_4_)F (chemical name sodium aluminium arsenate fluoride), has been determined previously [Kokkoros (1938). *Z. Kristallogr.* **99**, 38–49] using Weissenberg film data without reporting displacement parameters of atoms or a reliability factor. This study reports the redetermination of the structure of durangite using single-crystal X-ray diffraction data from a natural sample with composition (Na_0.95_Li_0.05_)(Al_0.91_Fe^3+^_0.07_Mn^3+^_0.02_)(AsO_4_)(F_0.73_(OH)_0.27_) from the type locality, the Barranca mine, Coneto de Comonfort, Durango, Mexico. Durangite is isostructural with minerals of the titanite group in the space group *C*2/*c*. Its structure is characterized by kinked chains of corner-sharing AlO_4_F_2_ octa­hedra parallel to the *c* axis. These chains are cross-linked by isolated AsO_4_ tetra­hedra, forming a three-dimensional framework. The Na^+^ cation (site symmetry 2) occupies the inter­stitial sites and is coordinated by one F^−^ and six O^2−^ anions. The AlO_4_F_2_ octa­hedron has symmetry -1; it is flattened, with the Al—F bond length [1.8457 (4) Å] shorter than the Al—O bond lengths [1.8913 (8) and 1.9002 (9) Å]. Examination of the Raman spectra for arsenate minerals in the titanite group reveals that the position of the band originating from the As—O symmetric stretching vibrations shifts to lower wavenumbers from durangite, maxwellite [ideally NaFe(AsO_4_)F], to tilasite [CaMg(AsO_4_)F].

## Related literature

For previous work on durangite, see: Brush (1869 ▶); Des Cloizeaux (1875 ▶); Kokkoros (1938 ▶); Machatschki (1941 ▶); Sumin de Portilla (1974 ▶); Foord *et al.* (1985 ▶). For minerals isostructural with or similar to durangite, see: Hawthorne (1990 ▶); Groat *et al.* (1990 ▶); Hawthorne *et al.* (1991 ▶); Oberti *et al.* (1991 ▶); Bermanec (1994 ▶); Cooper & Hawthorne (1995 ▶); Troitzsch *et al.* (1999 ▶); Sebastian *et al.* (2002 ▶). For Raman spectroscopic measurements on arsenate minerals and compounds, see: Yang *et al.* (2011*a* ▶,*b* ▶); Frost & Xi (2012 ▶); Frost *et al.* (2012 ▶). For the definition of polyhedral distortion, see: Robinson *et al.* (1971 ▶).

## Experimental

### 

#### Crystal data

(Na_0.95_Li_0.05_)(Al_0.91_Fe_0.09_)(AsO_4_)(F_0.73_OH_0.27_)
*M* *_r_* = 208.88
Monoclinic, 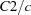

*a* = 6.5789 (5) Å
*b* = 8.5071 (6) Å
*c* = 7.0212 (5) Å
β = 115.447 (4)°
*V* = 354.83 (4) Å^3^
*Z* = 4
Mo *K*α radiation
μ = 10.18 mm^−1^
*T* = 293 K
0.10 × 0.10 × 0.09 mm

#### Data collection

Bruker APEXII CCD diffractometer
Absorption correction: multi-scan (*SADABS*; Sheldrick, 2005 ▶) *T*_min_ = 0.429, *T*_max_ = 0.461
2509 measured reflections
645 independent reflections
641 reflections with *I* > 2σ(*I*)
*R*_int_ = 0.019

#### Refinement

*R*[*F* ^2^ > 2σ(*F* ^2^)] = 0.013
*wR*(*F* ^2^) = 0.033
*S* = 1.10
645 reflections
44 parameters
3 restraints
H-atom parameters not refined
Δρ_max_ = 0.65 e Å^−3^
Δρ_min_ = −0.47 e Å^−3^

### 

Data collection: *APEX2* (Bruker, 2004 ▶); cell refinement: *SAINT* (Bruker, 2004 ▶); data reduction: *SAINT*; program(s) used to solve structure: *SHELXS97* (Sheldrick, 2008 ▶); program(s) used to refine structure: *SHELXL97* (Sheldrick, 2008 ▶); molecular graphics: *XtalDraw* (Downs & Hall-Wallace, 2003 ▶); software used to prepare material for publication: *publCIF* (Westrip, 2010 ▶).

## Supplementary Material

Crystal structure: contains datablock(s) I, global. DOI: 10.1107/S160053681204384X/wm2690sup1.cif

Structure factors: contains datablock(s) I. DOI: 10.1107/S160053681204384X/wm2690Isup2.hkl

Additional supplementary materials:  crystallographic information; 3D view; checkCIF report

## Figures and Tables

**Fig. 1.**
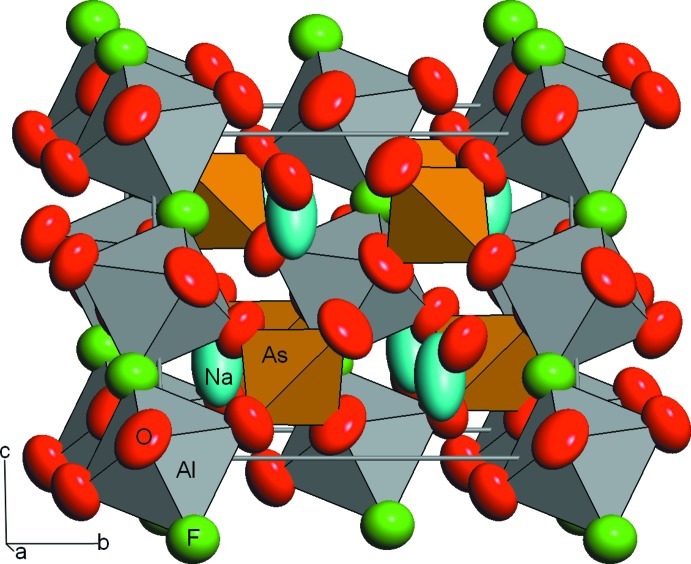
The crystal structure of durangite, NaAl(AsO4)F. The octahedra and tetrahedra represent the AlO4F2 and AsO4 groups, respectively.

**Fig. 2.**
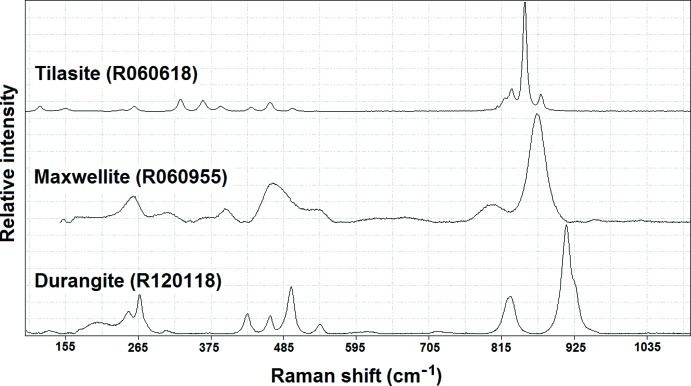
The Raman spectra of durangite, maxwellite, and tilasite. The spectra are shown with vertical offset for clarity.

## supplementary crystallographic information

### Comment

Durangite, NaAl(AsO_4_)F, is a member of the titanite mineral group crystallizing in space group *C*2/*c*, which includes a number of silicate, arsenate, phosphate, and sulfate minerals, as well as synthetic compounds with a wide range of chemical components (Hawthorne, 1990; Groat *et al.*, 1990; Sebastian *et al.*, 2002). Durangite was first described by Brush (1869) from tin placers in Durango, Mexico, and its crystallographic and optical data were measured by Des Cloizeaux (1875). Using Weissenberg photographs, Kokkoros (1938) determined its structure without reporting displacement parameters of atoms or a reliability factor. The synthesis of durangite was made by Machatschki (1941) and its infrared spectroscopic data were measured by Sumin de Portilla (1974). Foord *et al.* (1985) conducted a comprehensive mineralogical study on durangite from three different localities, including Black Range (New Mexico), Durango (Mexico), and Cornwall (England). Nevertheless, since the work by Kokkoros (1938), no further detailed crystallographic investigation has been reported for this mineral. As a part of our efforts to understand the crystal-chemical behavior of F *versus* OH in minerals, we concluded that the structural data for durangite need to be improved. This study reports a structure redetermination of durangite from the type locality by means of single-crystal X-ray diffraction.

Durangite is isostructural with minerals of the *C*2/*c* titanite group (*e.g.*, Hawthorne *et al.*, 1991; Oberti *et al.*, 1991; Troitzsch *et al.*, 1999) and topologically similar to the minerals of the *C*1 amblygonite (LiAlPO_4_F)-montebrasite (LiAlPO_4_OH) group (Groat *et al.*, 1990). Its structure is characterized by kinked chains of corner-sharing AlO_4_F_2_ octahedra (symmetry 1) running parallel to the *c* axis. These chains are cross-linked by isolated AsO_4_ tetrahedra, forming a three-dimensional framework. The Na^+^ cation (site symmetry 2) occupies the interstitial sites and is coordinated by one F and six O anions (Fig. 1). The AlO_4_F_2_ octahedron is flattened, with the Al—F bond lengths (1.8457 (4) Å) shorter than the Al—O bond lengths (1.8913 (9) and 1.9002 (9) Å). The average As—O, Al—(O,F), and Na—(O,F) bond lengths are 1.681, 1.879, and 2.420 Å, respectively.

In addition to durangite, two other arsenate minerals, namely maxwellite (ideally NaFe^3+^(AsO_4_)F) and tilasite (CaMg(AsO_4_)F), also belong to the *C*2/*c* titanite group. An examination of these arsenate mineral structures shows that the AsO_4_ tetrahedron appears to become increasingly distorted from durangite to maxwellite to tilasite, as measured by the tetrahedral angle variance (TAV) and quadratic elongation (TQE) indexes (Robinson *et al.*, 1971). The TAV and TQE values are 7.00 and 1.0018, respectively, for durangite, 9.80 and 1.0026 for maxwellite (Cooper & Hawthorne, 1995), and 15.45 and 1.0041 for tilasite (Bermanec, 1994). This observation may be correlated with the Ca content in these minerals, since our durangite sample shows no Ca, whereas the maxwellite sample examined by Cooper & Hawthorne (1995) contains 37% Ca substituting for Na.

Plotted in Figure 2 is the Raman spectrum for durangite, along with the Raman spectra for maxwellite and tilasite from the RRUFF Project (with RRUFF deposition numbers R060955 and R060618, respectively) for comparison. Note that our maxwellite sample is from the same locality as that studied by Cooper & Hawthorne (1995). Evidently, there are some resemblances among these Raman spectra. There have been numerous Raman spectroscopic studies on a variety of arsenate minerals and compounds (*e.g.*, Yang *et al.*, 2011*a*,*b*; Frost & Xi, 2012; Frost *et al.*, 2012, and references therein). In general, these spectra can be divided into three regions. Region 1, between 700 and 1000 cm^-1^, contains bands attributable to the As—O symmetric (the most intense band in each spectrum) and anti-symmetric stretching vibrations (ν_1_ and ν_3_ modes, respectively) within the AsO_4_ tetrahedra. Region 2, between 300 and 560 cm^-1^, includes bands originating from the O—As—O symmetric and anti-symmetric bending vibrations (ν_2_ and ν_4_ modes) within the AsO_4_ tetrahedron, overlapped with those from the *M*-(O,F) (*M* = octahedrally coordinated cations) stretching vibrations. The bands in Region 3, below 300 cm^-1^, are associated with the rotational and translational modes of AsO_4_ tetrahedra, as well as the O—M—O bending vibrations, Na-(O,F) interactions, and lattice vibrational modes.

One of the noticeable features in Figure 2 is that the position of the strongest band due to the As—O symmetric stretching vibrations is shifted to the lower wavenumbers from durangite (913 cm^-1^), maxwellite (870 cm^-1^), to tilasite (852 cm^-1^). This shift appears to be in line with the augmented distortion of the AsO_4_ tetrahedra from durangite, maxwellite, to tilasite, which, in turn, corresponds to the increased tilasite component in these minerals. Another visible feature in Figure 2 is the marked broadening of Raman bands for maxwellite relative to the corresponding ones for durangite and tilasite, indicating the strong short-range order of the maxwellite structure, resulting likely from its complex chemistry, i.e. (Na_0.56_Ca_0.41–0.03_)_Σ__=1_(Fe^3+^_0.24_Al_0.24_Fe^2+^_0.23_Mg_0.19_Ti_0.06_Mn_0.03_)_Σ__=1_(As_0.99_P_0.01_)_Σ__=1_O_4_)F_1.00_.

From chemical microprobe analysis, we estimated about 0.27 OH atoms per formula unit substituting for F in the structure. Unfortunately, we could not detect any obvious band attributable to the O—H stretching vibrations in the Raman spectra measurements. The possible hydrogen bond appears to be between F and O1, which are separated by a distance of 3.215 (1) Å.

### Experimental

The durangite crystal used in this study is from the type locality, the Barranca mine, Coneto de Comonfort, Durango, Mexico and is in the collection of the RRUFF project (http://rruff.info/R120118). Its chemical composition was measured with a CAMECA SX100 electron microprobe (9 analysis points), yielding the empirical chemical formula, calculated on the basis of 5 O atoms, (Li and OH were estimated by charge balance and difference).

The Raman spectra were collected from randomly oriented crystals at 100% power on a Thermo Almega microRaman system, using a solid-state laser with a wavenumber of 532 nm, and a thermoelectrically cooled CCD detector. The laser is partially polarized with 4 cm^-1^ resolution and a spot size of 1 µm.

### Refinement

Due to similar its X-ray scattering power, the small amount of Mn was treated as Fe during the refinement. Na and Li, Al and Fe, and F and the O atom of the OH group, respectively, were refined on the same sites and with the same displacement factors. All atomic sites were assumed to be fully occupied, yielding the structure formula (Na_0.95_Li_0.05_)(Al_0.91_Fe^3+^_0.09_)(As_1.00_O_4_)[F_0.73_(OH)_0.27_]. The highest residual peak in the difference Fourier maps was located at (0.0933, 0.3242, 0.3620), 0.77 Å from As, and the deepest hole at (0.0012, 0.3233, 0.3559), 0.74 Å from As.

### Crystal data

**Table:**

(Na_0.95_Li_0.05_)(Al_0.91_Fe_0.09_)(AsO_4_)(F_0.73_OH_0.27_)	*F*(000) = 395
*M**_r_* = 208.88	*D*_x_ = 3.915 Mg m^−^^3^
Monoclinic, *C*2/*c*	Mo *K*α radiation, λ = 0.71073 Å
Hall symbol: -C 2yc	Cell parameters from 2164 reflections
*a* = 6.5789 (5) Å	θ = 4.2–32.7°
*b* = 8.5071 (6) Å	µ = 10.18 mm^−^^1^
*c* = 7.0212 (5) Å	*T* = 293 K
β = 115.447 (4)°	Cuboid, red
*V* = 354.83 (4) Å^3^	0.10 × 0.10 × 0.09 mm
*Z* = 4	

### Data collection

**Table:**

Bruker APEXII CCD diffractometer	645 independent reflections
Radiation source: fine-focus sealed tube	641 reflections with *I* > 2σ(*I*)
Graphite monochromator	*R*_int_ = 0.019
φ and ω scan	θ_max_ = 32.6°, θ_min_ = 4.2°
Absorption correction: multi-scan (*SADABS*; Sheldrick, 2005)	*h* = −9→9
*T*_min_ = 0.429, *T*_max_ = 0.461	*k* = −12→12
2509 measured reflections	*l* = −10→10

### Refinement

**Table:**

Refinement on *F*^2^	Secondary atom site location: difference Fourier map
Least-squares matrix: full	H-atom parameters not refined
*R*[*F*^2^ > 2σ(*F*^2^)] = 0.013	*w* = 1/[σ^2^(*F*_o_^2^) + (0.0158*P*)^2^ + 0.3997*P*] where *P* = (*F*_o_^2^ + 2*F*_c_^2^)/3
*wR*(*F*^2^) = 0.033	(Δ/σ)_max_ < 0.001
*S* = 1.10	Δρ_max_ = 0.65 e Å^−^^3^
645 reflections	Δρ_min_ = −0.47 e Å^−^^3^
44 parameters	Extinction correction: *SHELXL97* (Sheldrick, 2008), Fc^*^=kFc[1+0.001xFc^2^λ^3^/sin(2θ)]^-1/4^
3 restraints	Extinction coefficient: 0.0311 (12)
Primary atom site location: structure-invariant direct methods	

### Special details

**Table:**

Geometry. All e.s.d.'s (except the e.s.d. in the dihedral angle between two l.s. planes) are estimated using the full covariance matrix. The cell e.s.d.'s are taken into account individually in the estimation of e.s.d.'s in distances, angles and torsion angles; correlations between e.s.d.'s in cell parameters are only used when they are defined by crystal symmetry. An approximate (isotropic) treatment of cell e.s.d.'s is used for estimating e.s.d.'s involving l.s. planes.
Refinement. Refinement of *F*^2^ against ALL reflections. The weighted *R*-factor *wR* and goodness of fit *S* are based on *F*^2^, conventional *R*-factors *R* are based on *F*, with *F* set to zero for negative *F*^2^. The threshold expression of *F*^2^ > σ(*F*^2^) is used only for calculating *R*-factors(gt) *etc*. and is not relevant to the choice of reflections for refinement. *R*-factors based on *F*^2^ are statistically about twice as large as those based on *F*, and *R*- factors based on ALL data will be even larger.

### Fractional atomic coordinates and isotropic or equivalent isotropic displacement parameters (Å^2^)

**Table:**

	*x*	*y*	*z*	*U*_iso_*/*U*_eq_	Occ. (<1)
Na	0.5000	0.17173 (9)	0.2500	0.01324 (16)	0.9500 (1)
Li	0.5000	0.17173 (9)	0.2500	0.01324 (16)	0.0500 (1)
Al	0.0000	0.0000	0.0000	0.00554 (10)	0.9100 (1)
Fe	0.0000	0.0000	0.0000	0.00554 (10)	0.0900 (1)
As	0.0000	0.314545 (18)	0.2500	0.00504 (7)	
O1	0.19850 (14)	0.43542 (11)	0.41553 (14)	0.00955 (16)	
O2	0.10283 (15)	0.20307 (10)	0.11309 (15)	0.00838 (15)	
F	0.0000	0.06705 (13)	−0.2500	0.00719 (18)	0.7300 (1)
OH	0.0000	0.06705 (13)	−0.2500	0.00719 (18)	0.2700 (1)

### Atomic displacement parameters (Å^2^)

**Table:**

	*U*^11^	*U*^22^	*U*^33^	*U*^12^	*U*^13^	*U*^23^
Na	0.0098 (3)	0.0063 (3)	0.0208 (4)	0.000	0.0039 (3)	0.000
Li	0.0098 (3)	0.0063 (3)	0.0208 (4)	0.000	0.0039 (3)	0.000
Al	0.00567 (19)	0.0052 (2)	0.00565 (19)	0.00045 (13)	0.00233 (15)	0.00008 (14)
Fe	0.00567 (19)	0.0052 (2)	0.00565 (19)	0.00045 (13)	0.00233 (15)	0.00008 (14)
As	0.00470 (9)	0.00455 (9)	0.00563 (9)	0.000	0.00199 (6)	0.000
O1	0.0072 (3)	0.0093 (4)	0.0103 (4)	−0.0026 (3)	0.0020 (3)	−0.0032 (3)
O2	0.0097 (3)	0.0071 (3)	0.0103 (4)	−0.0003 (3)	0.0061 (3)	−0.0020 (3)
F	0.0096 (4)	0.0067 (4)	0.0056 (4)	0.000	0.0036 (3)	0.000
OH	0.0096 (4)	0.0067 (4)	0.0056 (4)	0.000	0.0036 (3)	0.000

### Geometric parameters (Å, º)

**Table:**

Na—F^i^	2.2222 (14)	Al—O1^vii^	1.8913 (8)
Na—O2^ii^	2.3799 (9)	Al—O1^iii^	1.8913 (8)
Na—O2	2.3799 (9)	Al—O2^vi^	1.9002 (9)
Na—O1^iii^	2.4069 (11)	Al—O2	1.9002 (9)
Na—O1^iv^	2.4069 (11)	As—O1	1.6761 (8)
Na—O2^v^	2.5708 (10)	As—O1^viii^	1.6761 (8)
Na—O2^i^	2.5708 (10)	As—O2^viii^	1.6852 (9)
Al—F^vi^	1.8457 (4)	As—O2	1.6852 (9)
Al—F	1.8457 (4)		
			
F^vi^—Al—F	180.00 (6)	F—Al—O2	88.34 (4)
F^vi^—Al—O1^vii^	87.68 (3)	O1^vii^—Al—O2	90.23 (4)
F—Al—O1^vii^	92.32 (3)	O1^iii^—Al—O2	89.77 (4)
F^vi^—Al—O1^iii^	92.32 (3)	O2^vi^—Al—O2	180.00 (7)
F—Al—O1^iii^	87.68 (3)	O1—As—O1^viii^	104.31 (6)
O1^vii^—Al—O1^iii^	180.00 (8)	O1—As—O2^viii^	109.50 (5)
F^vi^—Al—O2^vi^	88.34 (4)	O1^viii^—As—O2^viii^	110.89 (4)
F—Al—O2^vi^	91.66 (4)	O1—As—O2	110.89 (4)
O1^vii^—Al—O2^vi^	89.77 (4)	O1^viii^—As—O2	109.50 (4)
O1^iii^—Al—O2^vi^	90.23 (4)	O2^viii^—As—O2	111.51 (6)
F^vi^—Al—O2	91.66 (4)		

Symmetry codes: (i) −*x*+1/2, −*y*+1/2, −*z*; (ii) −*x*+1, *y*, −*z*+1/2; (iii) −*x*+1/2, *y*−1/2, −*z*+1/2; (iv) *x*+1/2, *y*−1/2, *z*; (v) *x*+1/2, −*y*+1/2, *z*+1/2; (vi) −*x*, −*y*, −*z*; (vii) *x*−1/2, −*y*+1/2, *z*−1/2; (viii) −*x*, *y*, −*z*+1/2.
